# Emerging Structural Insights into Glycoprotein Quality Control Coupled with *N*-Glycan Processing in the Endoplasmic Reticulum

**DOI:** 10.3390/molecules20022475

**Published:** 2015-01-30

**Authors:** Tadashi Satoh, Takumi Yamaguchi, Koichi Kato

**Affiliations:** 1 Graduate School of Pharmaceutical Sciences, Nagoya City University, 3-1 Tanabe-dori, Mizuho-ku, Nagoya 467-8603, Japan; E-Mail: takumi@ims.ac.jp; 2 JST, PRESTO, 3-1 Tanabe-dori, Mizuho-ku, Nagoya 467-8603, Japan; 3 Okazaki Institute for Integrative Bioscience and Institute for Molecular Science, National Institutes of Natural Sciences, 5-1 Higashiyama, Myodaiji, Okazaki, Aichi 444-8787, Japan

**Keywords:** calnexin/calreticulin cycle, cargo receptor, endoplasmic reticulum, intracellular lectin, *N*-glycan processing, NMR spectroscopy, glycoprotein quality control, X-ray crystallography

## Abstract

In the endoplasmic reticulum (ER), the sugar chain is initially introduced onto newly synthesized proteins as a triantennary tetradecasaccharide (Glc_3_Man_9_GlcNAc_2_). The attached oligosaccharide chain is subjected to stepwise trimming by the actions of specific glucosidases and mannosidases. In these processes, the transiently expressed *N*-glycans, as processing intermediates, function as signals for the determination of glycoprotein fates, *i.e.*, folding, transport, or degradation through interactions of a series of intracellular lectins. The monoglucosylated glycoforms are hallmarks of incompletely folded states of glycoproteins in this system, whereas the outer mannose trimming leads to ER-associated glycoprotein degradation. This review outlines the recently emerging evidence regarding the molecular and structural basis of this glycoprotein quality control system, which is regulated through dynamic interplay among intracellular lectins, glycosidases, and glycosyltransferase. Structural snapshots of carbohydrate-lectin interactions have been provided at the atomic level using X-ray crystallographic analyses. Conformational ensembles of uncomplexed triantennary high-mannose-type oligosaccharides have been characterized in a quantitative manner using molecular dynamics simulation in conjunction with nuclear magnetic resonance spectroscopy. These complementary views provide new insights into glycoprotein recognition in quality control coupled with *N*-glycan processing.

## 1. Introduction

*N*-linked glycans as biological signal molecules are characterized by their intricate structures in comparison with other biosignal modifiers such as phosphate and sulfate. *N*-glycans have branched structures with extremely high-degrees of freedom in internal motions. This structural feature enables the glycans to embed and express multiple messages deciphered by various lectins, which are well exemplified by high-mannose-type oligosaccharides, as fate determinants of glycoproteins in the early secretory pathway.

**Figure 1:**
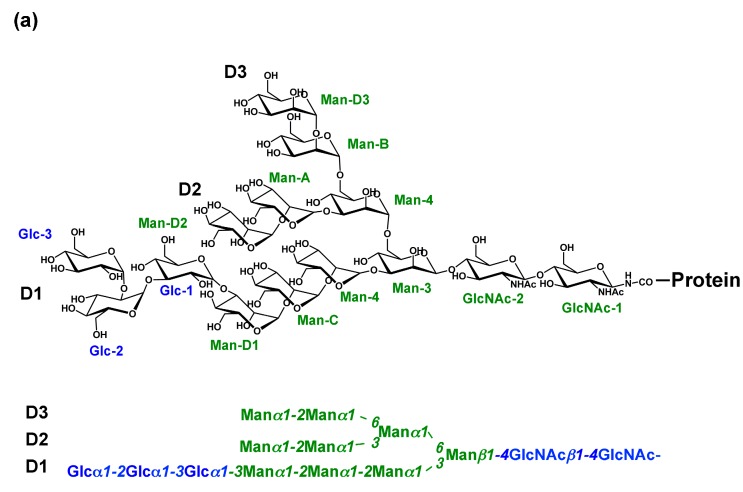

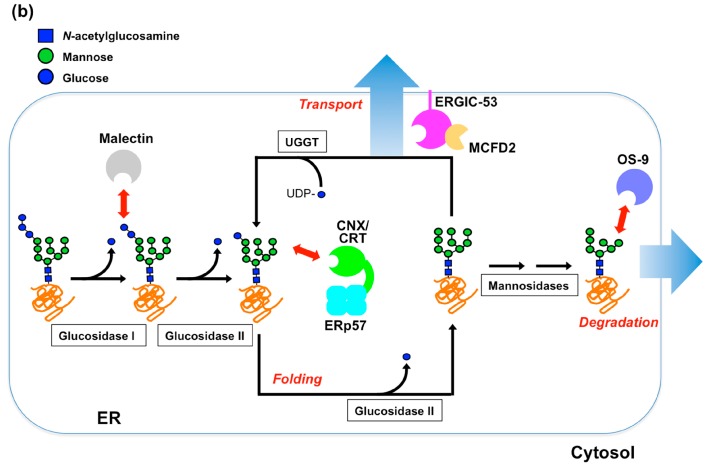
(**a**) Schematic representation of Glc_3_Man_9_GlcNAc_2_ indicating the nomenclature of oligosaccharide residues and branches according to the custom stated by Vliegenthart *et al*. [1]; (**b**) Scheme of *N*-glycan-dependent fate determination of glycoproteins in the ER. Glycoprotein-fate determination couples with *N*-glycan processing through interactions with a series of intracellular lectins.

In the endoplasmic reticulum (ER), newly synthesized proteins are modified with tetradecasaccharide (Glc_3_Man_9_GlcNAc_2_), which harbors three nonreducing terminal branches (termed D1, D2, and D3) [1], one of which (D1) is capped with the triglucosyl moiety Glc-α1,2-Glc-α1,3-Glc (Figure 1a). The biological messages leading to folding, translocation, and degradation of the glycoproteins are concealed within this triantennary structure and transiently expressed during *N*-glycan processing for the determination of the glycoprotein fate by a series of intracellular lectins (Figure 1b) [2,3,4,5]. Recently, new crystal structures have been reported for several key players in this system. Furthermore, a combined approach of nuclear magnetic resonance (NMR) spectroscopy and molecular dynamics (MD) simulation has provided dynamic views of *N*-glycan conformations in solution. This review will outline the emerging insights into the molecular mechanisms underlying the *N*-glycan-mediated protein quality control on the basis of these new structural data.

## 2. Glycoprotein Folding Assisted by Lectin/Chaperone Complexes

The initial step of the *N*-glycan processing in the ER is the removal of the non-reducing terminal glucose residue at the D1 branch. Glucosidase I is responsible for this unsealing process by catalyzing the cleavage of the D1 terminal Glc-α1,2-Glc glycosidic linkage [6,7]. This enzyme is a type II transmembrane protein of 80 kDa–110 kDa, possessing a large catalytic domain (belonging to the glycoside hydrolase (GH) family 63). The recently reported crystal structure of yeast glucosidase I, Cwh41p [8], shows a globular structure comprising a 32-kDa N-terminal β-sandwich domain and a 57 kDa C-terminal (α/α)_6_ toroid domain, which contains catalytic residues. Structure-based mutagenesis experiments and docking simulations have led to the proposal that the specificity of this enzyme is governed by a unique conformation of the glucotriose substrate, *i.e.*, a bentback conformation with an intrachain interaction between the first and the third glucose residues [8]. It has been proposed that the suppression of glucosidase I activity leads to the decreased infectivity of several viruses, including HIV and influenza viruses, because most viral envelope glycoproteins contain *N*-linked oligosaccharides [9,10,11]. In this context, the structural information is helpful in the design of specific and potent inhibitory compounds that could be developed as antiviral drugs.

**Figure 2:**
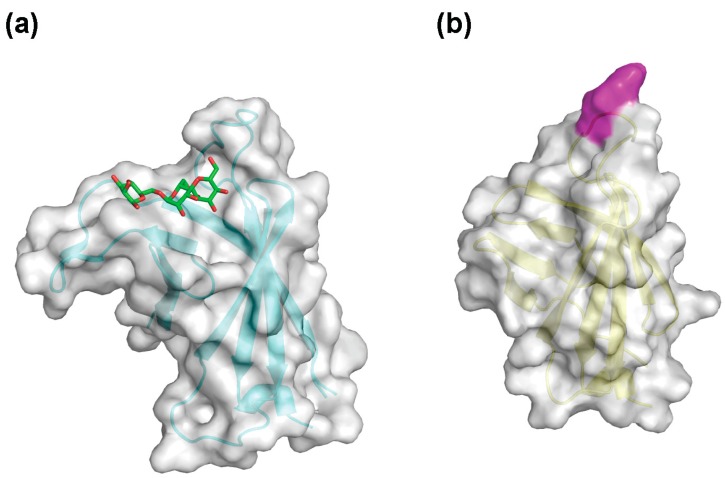
Transparent surface models of the MRH domains of OS-9 (PDB code: 3AIH) [18] and glucosidase II β subunit (PDB code: 2LVX) [17] are shown in (**a**,**b**), respectively. Bound α1,6-linked mannotriose in the crystal structure of the MRH domain of OS-9 is shown as stick models. Residues interacting with the M9 oligosaccharide evaluated using NMR data of the MRH domain of the glucosidase II β subunit are colored in magenta. Trp409 is colored in orange.

The cleavages of the second and third glucose residues, both of which are connected through α1,3-linked glycosidic linkages, are catalyzed by the same enzyme glucosidase II [7,12,13,14], although the cleavage at the Glc-α1,3-Glc linkage is catalyzed more efficiently than that at the Glc-α1,3-Man linkage [13,14]. This enzyme comprises approximately a 110-kDa catalytic α subunit and a 60-kDa regulatory β subunit. The α subunit is predicted to belong to the GH31 family and adopts a globular structure, while the β subunit contains a mannose-6-phosphate receptor homology (MRH) domain and a flexible, extended region, which has been suggested on the basis of limited proteolysis and ultracentrifugation analyses [15]. The previous frontal affinity chromatography (FAC) data demonstrated that the MRH domain in the β subunit has a higher affinity for oligosaccharides containing α1,2-linked mannobiose structure on the D3 branch (Figure 1a) [16]. A recently reported NMR study has determined the solution structure of the isolated MRH domain and successfully identified the binding site of Man_9_GlcNAc_2_ (M9) and α1,2-linked mannobiose [17], indicating that the sugar-binding pocket is shallower than that of the other MRH domain, including OS-9, which is involved in ER-associated degradation (ERAD) [18] (Figure 2). The authors identified a conserved tryptophan (Trp409) as a key residue, which is located outside the binding pocket but affects enzyme activity [17]. The cleavage of the Glc-α1,3-Glc linkage at the D1 branch is catalyzed by the α subunit, giving rise to the determinant recognized by the ER chaperones, while the mannosidase-catalyzed cleavage of the Man-α1,2-Man at the D3 branch, the primary glycotope recognized by the β subunit, is a key step leading to ERAD (see below). Therefore, the cooperation between the α and β subunits of glucosidase II is likely to be a critical mechanism in the processes of glycoprotein fate determination. Hence structural information is desirable for understanding the substrate-binding mode of the α/β heterodimeric form of this enzyme.

The monoglucosylated D1 branch, which is transiently displayed between the second and the third glucose-trimming steps catalyzed by glucosidase II, serves as a specific determinant recognized by the ER resident lectins, *i.e.*, type I membrane protein calnexin (CNX) and its soluble homolog calreticulin (CRT) [3,19,20]. These lectins comprise a globular carbohydrate recognition domain (CRD) with a structural resemblance to legume lectins and an extended proline-rich segment called P-domain, which recruits ERp57, a protein disulfide isomerase (PDI) family chaperone [21,22,23,24,25], and thereby assists in the folding of the nascent glycoproteins. Beside this canonical lectin/chaperone complex, another PDI family protein, ERp29, has recently been reported to form a 1:1 complex with CRT [26]. Unlike ERp57, which possesses two catalytic thioredoxin (Trx)-like domains, ERp29 has a single Trx-like domain without the CXXC catalytic motif, indicating that this protein is not directly involved in thiol/disulfide exchange reactions. The crystallographic data of CRT^CRD^ in complex with Glc-α1,3-Man-α1,2-Man-α1,2-Man, which corresponds to the monoglucosylated D1 branch, revealed a distinct interaction mode as compared with legume-type (l-type) lectins; despite their overall structural similarities, the monoglucosylated D1 branch is accommodated in parallel with β-strands on the concave β-sheet of CRT^CRD^, whereas mannosyl ligands are accommodated in crossing β-strands in l-type lectins including ERGIC-53^CRD^ and VIP36^CRD^ [22,27,28,29]. Structural similarity and conservation of oligosaccharide-binding residues between the CRD domains of CRT and CNX suggest essentially common interaction modes of the monoglucosylated D1 shared by these two ER lectins.

**Figure 3:**
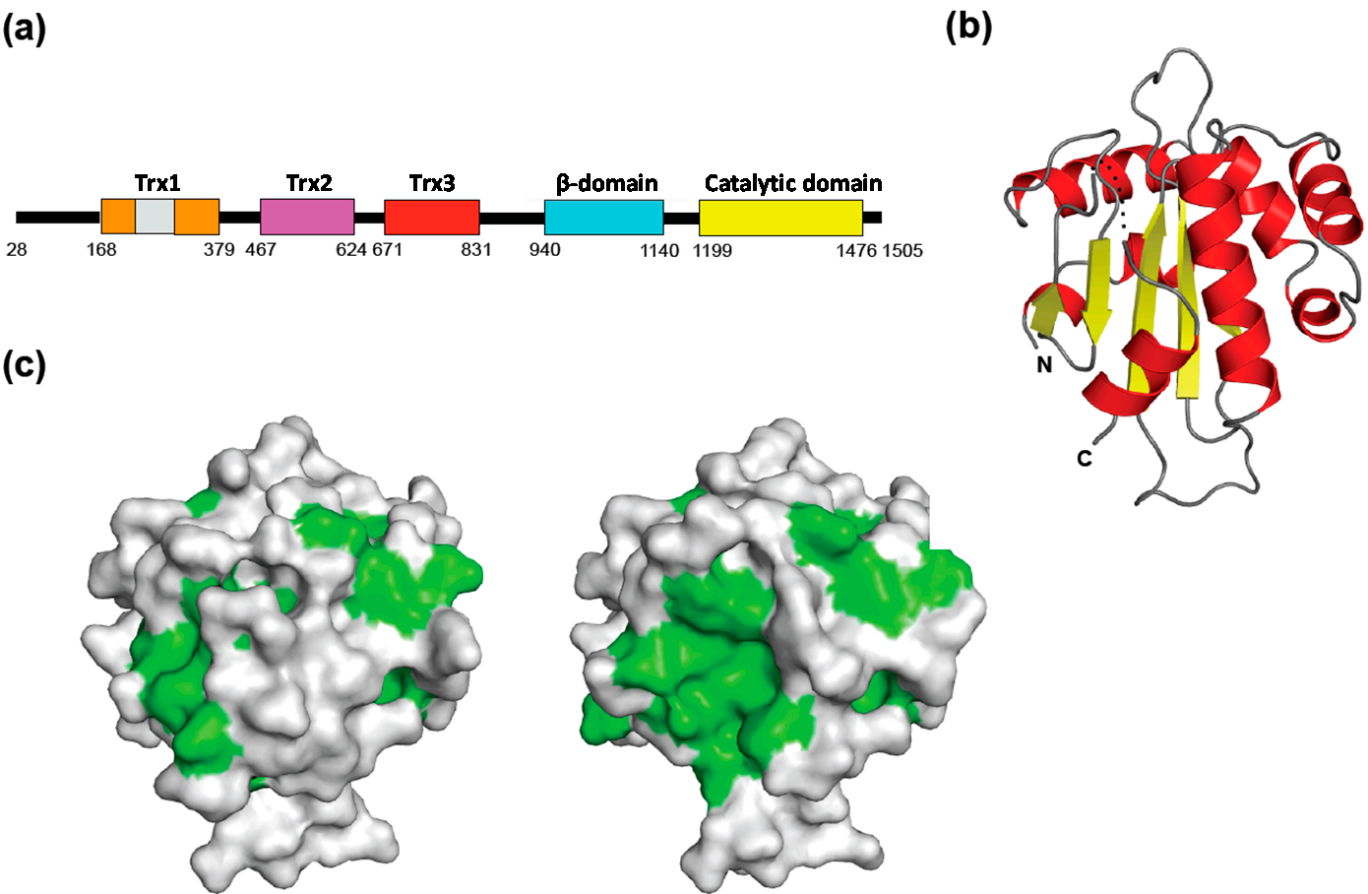
(**a**) Domain architecture of the thermophilic fungal UGGT; (**b**) Ribbon models of the third Trx domain of the *Chaetomium thermophilum* UGGT (closed form, PDB code: 3WZT) [33]. The secondary structures are highlighted (α-helix, red; β-sheet, yellow), while the linker regions are indicated in gray. Dotted line indicates disordered segment; (**c**) Surface models of the closed and open (PDB code: 3WZS) forms of the Trx3 domain are shown on the left and right, respectively. The hydrophobic residues are shown in green.

This CNX/CRT-mediated folding assistance system is equipped with an elaborate backup mechanism, *i.e.*, the folding sensor enzyme UDP-glucose:glycoprotein glucosyltransferase (UGGT), which provides the already deglucosylated yet incompletely folded glycoproteins with another attempt to interact with the lectin/chaperone complex by regenerating its monoglucosylated glycoforms [12,19,30,31,32]. These glucose-trimming and -tagging processes constitute the CNX/CRT cycle. UGGT serves as a gatekeeper in this surveillance system because this enzyme is capable of sensing the folding states of glycoproteins as potential substrates [30,31,32]. Although the C-terminal domain (accounting for 20% of amino acid sequence) has been predicted to belong to the glycosyltransferase 8 family, no structural information was available for the putative folding sensor region (accounting for the remaining 80% of amino acid residues) of this key enzyme until recently. Our bioinformatics analyses suggested that the folding-sensor region of UGGT contains three tandem Trx-like domains, which are often found in ER-resident proteins that are involved in protein quality control [33] (Figure 3a). UGGT can form a complex with Sep15, a 15-kDa selenocystein-containing oxidoreductase possessing one redox-active Trx-like domain. Although Sep15 binding did not affect UGGT-catalyzed glucosylation of a model glycoprotein substrate, such as denatured thyroglobulin [34], the enzymatic activity of UGGT was enhanced upon this complex formation, as revealed when probed with a synthetic substrate comprising Man_9_GlcNAc_2_ (M9) and a hydrophobic fluorescent dye [35]. It is conceivable that Sep15 serves as a structural and functional extension of UGGT. Furthermore, our recently reported crystal structures of the third Trx-like domain of thermophilic fungal UGGT show its extensive hydrophobic patch, which is masked by the flexible C-terminal helix in a closed conformation, whereas it is capable of trapping a hydrophobic molecule through the exposed hydrophobic patch in an open conformation (Figure 3b,c) [33]. These results provide a clue to the mechanism of the manner in which UGGT senses the folding states of potential substrates in the ER quality control system.

## 3. Glycoprotein Transport Mediated by Lectins as Cargo Receptors

Correctly folded glycoproteins that successfully passed through the CNX/CRT surveillance system in the ER are transported to the Golgi complex via the ER–Golgi intermediate compartment (ERGIC). During intracellular trafficking, sorting and loading of the cargo glycoproteins into the transport vesicles are mediated by transmembrane lectins, operating as receptors, including ERGIC-53 and VIP36 [2,3,4,5]. These receptors are categorized as l-type lectins because they possess homologous CRDs with structural similarity to legume lectins such as concanavalin A. Previous FAC and structural data revealed that they exhibit disparate sugar-binding specificities and affinities, despite structural similarities in their CRDs, suggesting their distinct biological roles in intracellular trafficking [36,37]. VIP36 specifically recognizes a nonglucosylated α1,2-linked D1 trimannosyl structure. In contrast, ERGIC-53 exhibits a broader specificity towards the high-mannose-type glycans, irrespective of the presence or absence of the terminal monoglucose residue at the D1 branch, which corresponds to the aforementioned determinant recognized by the CNX/CRT lectins. Expression of ERGIC-53 together with several ER chaperones is upregulated under ER stress conditions, implying that this lectin facilitates the export capacity of glycoprotein cargos under emergency conditions [37,38,39,40]. The glycoproteins that remain incompletely folded but anterogradely translocated with monoglucosylated high-mannose-type glycans may be passed back to the ER by ERGIC-53 but not VIP36. Alternatively, it is possible that the monoglucosylated glycans can be trimmed by action of Golgi endo-α-mannosidase [41] and subsequently digested by Golgi exo-α-mannosidases for further processing [42].

**Figure 4:**
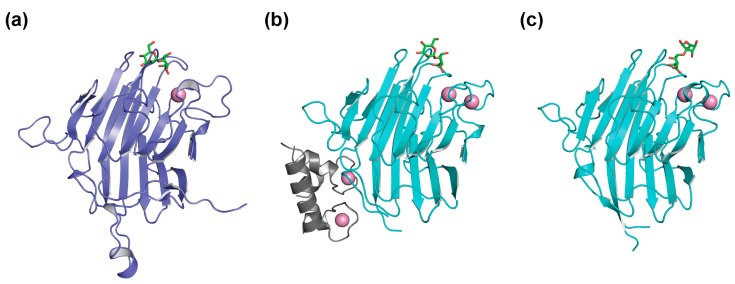
Ribbon models of the sugar-bound complexes of (**a**) VIP36^CRD^ (PDB code: 2DUR) [27]; (**b**) ERGIC-53^CRD^–MCFD2 (PDB code: 3WNX) [29]; and (**c**) ERGIC-53^CRD^ (PDB code: 4GKX) [28]. Bound α1,2-linked mannobiose in the complex structures is represented in stick models.

Cumulative crystallographic data have provided the structural basis for the different sugar-binding specificities of the homologous l-type lectins, ERGIC-53 and VIP36, at the atomic level. Regarding VIP36, we previously determined the crystal structures complexed with mannosyl ligands, and thereby revealed that the nonglucosylated D1 branch is accommodated on the concave β-sheet of its CRD, thus indicating the underlying mechanisms of its sugar-binding specificity [27] (Figure 4a). Very recently, two independent groups reported crystal structures of ERGIC-53^CRD^ complexed with sugar ligands [28,29] (Figure 4b,c).

**Figure 5:**
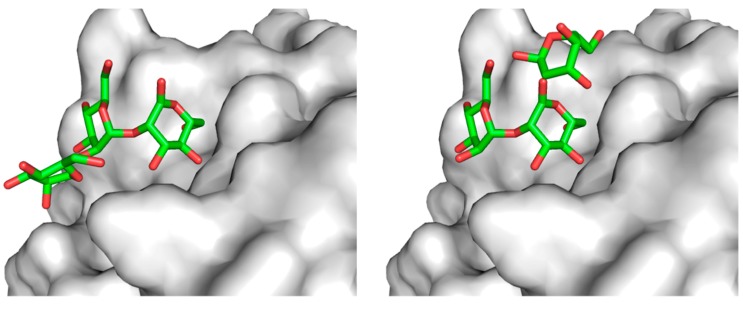
Two alternative modes for interactions of α1,2-linked mannotriose with ERGIC-53^CRD^. The terminal nonreducing (**left**) and reducing (**right**) mannose residues were modeled, as previously described [29].

Zheng and coworkers reported the structure of ERGIC-53^CRD^ in complex with Man-α1,2-Man [28], whereas we determined the structure of the ternary complex comprising ERGIC-53^CRD^, its binding partner, MCFD2, which is a 16-kDa Ca^2+^-binding protein, and the Man-α1,2-Man-α1,2-Man, which corresponds to the entire D1 branch [29]. The crystallographic data indicate that the D1 trimannosyl branch is accommodated in ERGIC-53^CRD^ exhibiting two alternative modes of interactions. One mode is similar to that of VIP36^CRD^, which, however, has a protruding aspartate residue instead of glycine at the corresponding position of the sugar-binding pocket of ERGIC-53^CRD^, rendering the orientation of the nonreducing terminal mannose residue (corresponding to Man-D1) to be significantly altered. This allows ERGIC-53^CRD^ to accommodate the monoglucosylated D1 branch (Figure 5a). In the other interaction mode, the 3-OH group of the nonreducing terminal mannose residue is positioned outward against the sugar-binding pocket, also permitting the α1,3-linked monoglucosylation without steric hindrance (Figure 5b). Such alternative sugar-binding modes in recognition of the identical mannosyl ligand have been reported for concanavalin A [43]. These findings have provided a structural basis for the broad sugar-binding specificity of the ERGIC-53 lectin.

The blood coagulation factors V and VIII are two of the most extensively studied cargo glycoproteins employing the ERGIC-53–MCFD2 complex as a cargo receptor in the secretory pathway [44,45,46,47]. On the basis of the structural data of ERGIC-53^CRD^ and MCFD2, along with their complex, a mechanistic model has been proposed, which involves a conformational change of MCFD2 with the opening of its putative ligand-binding pocket upon complex formation with the CRD [48,49].

In this model, ERGIC-53 interacts with the *N*-linked sugar chains of these cargo glycoproteins, whereas MCFD2 captures their polypeptide segments. Recently, new cargo glycoproteins were identified for ERGIC-53, including Mac-2BP, which is a large oligomeric secretory glycoprotein and interacts with ERGIC-53 in an MCFD2-dependent manner [50]. ERGIC-53 is also shown to be required for the production of infectious arenavirus, coronavirus, and filovirus particles [51]. ERGIC-53 interacts with the envelope glycoproteins of these viruses through its CRD; however, their association was mediated by neither the viral glycans nor MCFD2.

## 4. Glycoprotein Degradation Mediated by ERAD Lectins

In contrast to the folding and transport systems, terminally misfolded proteins are translocated from the ER into the cytosol for ERAD, which is mediated by the ubiquitin–/proteasome-mediated proteolytic system. ER-degradation enhancing α-mannosidase-like proteins (EDEMs) and their yeast homolog Htm1p were originally identified as lectins that recognize Man_8_GlcNAc_2_ isomer B (M8B) and facilitate glycoprotein ERAD [52,53]. However, there has been a controversy as to whether the EDEM family proteins function as mannosidases or lectins. Although the crystal structures of ER mannosidase I and its yeast homolog Mns1p have been determined [54,55,56,57], no structural information regarding EDEMs has thus far been available. Recently accumulated evidence indicated that the EDEM family proteins exert mannosidase activities, which is involved in generation of α1,6-linked glycotope at the D3 branch [58,59,60,61,62,63,64].

Although it was suggested that the Htm1p-Pdi1p complex, like UGGT, discriminate the folding state of glycoprotein substrates [65], the question as to whether the EDEM family proteins catalyze mannose trimming selectively against misfolded glycoproteins still remains elusive. A plausible alternative explanation for the selective degradation of terminally misfolded glycoproteins is that mannose trimming serves as a countdown timer of the window time frame that is available for glycoprotein folding in the ER [66].

The exposed α1,6-linked glycotope generated by the action of EDEMs (yeast homolog Htm1p) is recognized by OS-9 (yeast homolog Yos9p), which possesses an MRH lectin domain similar to the β subunit of glucosidase II. The Man-α1,6-linked D3 branch displayed on potential ERAD substrates is inspected by the OS-9 MRH domain; this is a crucial step for guiding the misfolded proteins into ERAD [60,61,63,67]. OS-9 is tethered to the SEL1L-contaning ER membrane dislocation complex presumably through its interaction with *N*-linked glycans displayed on SEL1L [18,68,69]. In contrast to OS-9, the functional role of its homolog XTP3-B, containing two homologous MRH domains, remains controversial [70,71]. While flow cytometry data suggested that the C-terminal MRH domain interacts with *N*-glycans bearing α1,6-linked glycotope [70], FAC analyses detected only marginal affinity of XTP3-B to the M9 oligosaccharide. Furthermore, unlike OS-9, XTP3-B did not facilitate degradation of misfolded glycoprotein substrates; however, instead inhibited ERAD of the misfolded glycoproteins exhibiting M9 glycans [71].

Our structural studies demonstrated that the MRH domain of OS-9 interacts with the Man-α1,6-Man-α1,6-Man residues on the D3 branch of the high-mannose-type oligosaccharide, thereby providing a structural basis for the sugar-binding specificity of this lectin [18]. In this structure, the outermost mannose (corresponding to Man-B) is just accommodated on its small pocket, obviously rejecting the mannosyl branch having an additional α1,2-linked mannose residue (corresponding to Man-D3) because of steric hindrance (Figure 2a). Our finding coincided with the fact that removal of Man-D3 by EDEM1/3 is a prerequisite for glycoprotein ERAD. As in the case of structural studies on the l-type lectins [27,28,29], this crystal structure along with the NMR structure of the MRH domain of the glucosidase II β subunit, has given insights into structural mechanisms of the manner in which structurally homologous MRH domains exhibit disparate sugar-binding specificities in terms of the presence of the outermost mannose residues at the D3 branch.

In addition, the sensing ability of the glycoprotein folding states has been suggested for OS-9/Yos9p [62,68,69]. However, it remains elusive whether the ERAD lectins directly recognize folding states of potential ERAD substrates or whether they simply act as lectins in cooperation with ER chaperones such as BiP and GRP94. Recently, two independent groups reported the *in vitro* interaction mode between OS-9 and GRP94 and the *in vivo* function of their complex [72,73]. In the former report, the disordered C-terminal segment of OS-9, including a mammalian-specific insertion following the MRH domain, was involved in the interaction over the middle and C-terminal domains of GRP94, suggesting cooperative interplay between these two proteins in the motionally flexible complex [72]. The latter report showed that GRP94 did not cooperate with OS-9 in ERAD of misfolded substrates. It has been alternatively proposed that OS-9 associates preferentially with a minor population of GRP94, which is hyperglycosylated on its cryptic *N*-linked glycan acceptor sites, thereby facilitating the turnover of nonnative GRP94 [73]. To date, structural data of the ERAD factors have only been available for the MRH domain of OS-9 and the dimerization domain of Yos9p [18,74]. To obtain deeper insights into substrate selection mechanisms in ERAD, structural information is highly desired in terms of molecular recognition by the ERAD players including EDEMs.

## 5. Conformational Dynamics of High-Mannose-Type Oligosaccharides

As described in the sections above, crystallographic studies have successfully provided conformational snapshots of oligosaccharides recognized by lectins, thereby offering a structural basis for their sugar-binding specificities. However, these data give only one-sided views of glycan recognition mechanisms in terms of the energetics of carbohydrate-protein interactions, which have to be characterized considering the hydration and conformational states of glycans and lectins in their uncomplexed forms. In particular, glycans are supposed to have considerable conformational flexibility, at least in their uncomplexed states. Therefore, carbohydrate-protein interactions generally involve the loss of conformational entropy, which significantly contributes to the free energy difference between the free and bound states of interaction systems [75]. The triantennary high-mannose-type oligosaccharides possess remarkable degrees of motional freedom; therefore, they occupy vast conformational spaces.

Dynamic three-dimensional structures of high-mannose-type oligosaccharides in solution have been characterized by employing computational techniques such as molecular mechanics and dynamics simulations and NMR spectroscopic measurements inspecting scalar coupling, nuclear Overhauser effect, and paramagnetic relaxation enhancement [76,77,78,79,80,81]. Moreover, we have recently developed a methodology to explore the conformational spaces of highly branched oligosaccharides by combining MD simulations on the basis of a generalized-ensemble algorithm combined with a paramagnetism-assisted NMR technique [82,83].

**Figure 6:**
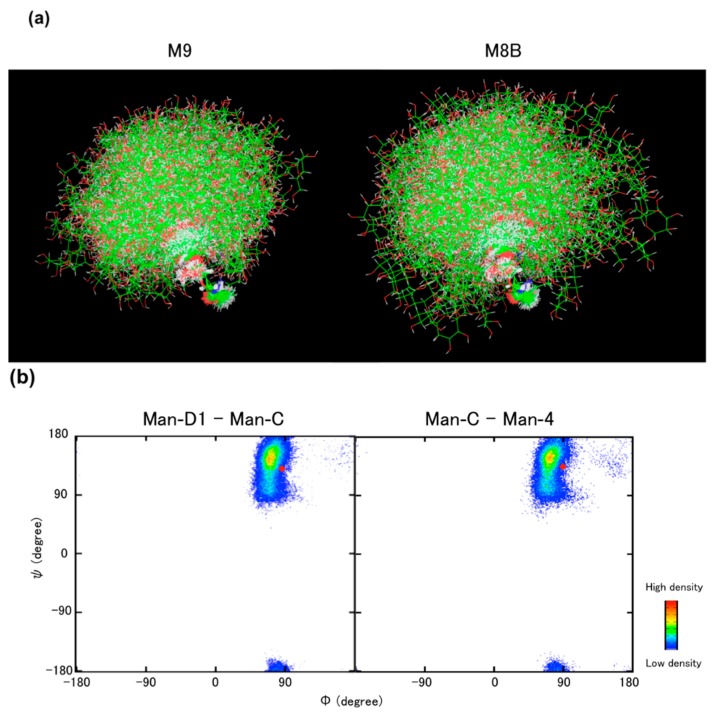
(**a**) Superimposition of 240 conformers extracted from the replica exchange MD simulation results of the M9 and M8B oligosaccharides [84]; (**b**) Density maps of glycosidic linkage torsion angles of the α1,2-linked trimannosyl moiety at the D1 branch of the M9 oligosaccharide obtained using NMR-validated replica exchange MD simulations. Red circles indicate the conformations of the Man-α1,2-Man glycosidic linkages of the sugar chain in complex with VIP36^CRD^, (Φ, ψ) = (89°, 132°) and (92°, 134°) corresponding to the Man-D1–Man-C and Man-C–Man-4 linkages, respectively, on the basis of crystallographic data (PDB codes: 2DUR and 2E6V, respectively) [27]. The torsion angles Φ and ψ are defined as O_5_-C_1_-O_1_-C'_2_ and C_1_-O_1_-C'_2_-C'_3_, respectively. Figure 6a was adapted from Yamaguchi *et al*. [84] with the permission of John Wiley and Sons.

The experimentally validated simulations have allowed us to describe the dynamic conformational ensemble of the M9 oligosaccharide; it exhibits foldback conformations in which the outer mannose residues in the D2 and D3 branches are in spatial proximity to the reducing terminus, while the D1 branch remains distal thereto [84] (Figure 6a). The conformation of the α1,2-linked D1 trimannosyl moiety, accommodated in VIP36^CRD^, which was determined on the basis of crystallographic data, corresponds to a low-populated conformational state of this branch among its conformational ensemble, which was derived from the NMR-validated MD simulation (Figure 6b). These data suggest conformational selection by the lectin during the glycan recognition process. Furthermore, comparison of the MD simulations between the M9 and M8B oligosaccharides indicated that the removal of the nonreducing terminal mannose residue at the D2 branch, *i.e.*, Man-D2, results in significant expansion of the conformational space with increased population of the foldback conformations (Figure 6a). These findings imply that outer carbohydrate trimming not only exposes the embedded glycotopes as fate determinants but also affects the conformational space of the remaining part of the triantennary *N*-glycans. Such dynamic conformational rearrangements of high-mannose-type oligosaccharides can be important factors in inspecting glycan-binding affinities and specificities of the lectins and enzymes involved in the determination of glycoprotein fate in the ER.

## 6. Concluding Remarks and Perspectives

Carbohydrate recognition is controlled by static steric factors such as the shape of the sugar-binding pocket of lectin and non-reducing terminal capping of glycans. In addition to this static view, dynamic aspects are critically important for better understanding of carbohydrate-lectin interactions, as exemplified by the multiple ligand-binding modes of ERGIC-53 and the dynamic conformational ensembles of triantennary high-mannose-type oligosaccharides. The ER quality control system is ingeniously designed by combining these mechanisms employing the high-mannose-type glycans as transformable signals recognized by a series of intracellular lectins and processing enzymes, which exert distinct ligand specificities despite often homologous structural frameworks. To provide physicochemical basis of specificities and affinities of these lectins and enzymes, energy landscapes of the carbohydrate recognition systems should be quantitatively characterized, taking account of dynamic behaviors of proteins, oligosaccharides, and water molecules under molecular crowding conditions. Multilateral experimental approaches in conjunction with theoretical approaches will be obviously needed to gain further insights into glycoprotein quality control coupled with *N*-glycan processing in the ER.
